# 

**Published:** 1824-08

**Authors:** 


### MONTHLY CATALOGUE OF MEDICAL BOOKS.

⁂ *It having been hinted to us that gentlemen, sending copies of their Works, prefer having their titles given at length, it is our intention, in future, to comply with this suggestion: but it is to be observed, that no books can be entered on this List except those sent to us for the purpose;—finding, in the lists hitherto transmitted, that the names of works have frequently been given as published, which have not appeared for weeks, or even months after.*

Pathological Views of the Structure, Functions, and Disorders of the Stomach and Alimentary Organs of the Human Body; with Observations on the Qualities and Effects of Food and Fermented Liquors, and on the Influence of Climate and local Situation. By THOMAS HARE, F.L.S. F.H.S. Fellow of the Royal College of Surgeons in London, &c. Second Edition.—Pp. 368. London, 1824.

Inductions Physiologiques, Pathologiques, et Therapeutiques; ou Elemens géneraux d'Anthropologie et de Medecine, deduits des Faits. Précedés d'un Précis historique des Doctrines Anthropologiques et Medicales. Par J. F. COFFIN.—Pp. 175. Paris, 1822.

A Voyage to India; containing Reflections on a Voyage to Madras and Bengal, in 1821, in the Ship Lomach; Instructions for the Preservation of Health in Indian Climates; and Hints to Surgeons and Owners of Private Trading Ships. By JAMES WALLACE, Surgeon of the Lomach.—Pp. 166. London, 1824.

Competitio ad Aggregationem Jussu Regis optimi et ex Mandato Summi Regiæ Universitatis Magistri instituta 1823. An in Curanda Oculi Suffusione (vulgo Cataracte), Lentis Crystallinæ Extractio hujus Depressione præstantior? Theses, quas Deo favente, in saluberrima Facultate medica Parisiensi, præsentibus competitionis judicibus, publicis competitorum disputationibus subjiciet et dilucidare conabitur die 26 Februarii, anno 1824. Auctor J. CLOQUET.—Pp. 9. Parisiis, 1824.

Consultations et Observations de Médecine de feu C. L. DUMAS, Recteur de l'Academie de Montpelier, Doyen et Professeur d'Anatomie et de Physiologie de la Faculté de Medecine de la même Ville; Professeur de Clinique de perfectionnement appliquée aux Maladies Chroniques; et Medecin de l'Hospice établi pour le Traitement de ses Maladies; Correspondant de l'Institut de France, &c. Publiées par le Dr. ROUZET, Membre adjoint de l'Academie Royale de Médecine, &c. &c.—Pp. 498. Paris, 1824.

Melanges de Chirurgie Etrangère, par une Societé de Chirurgiens de Genève, composée de MM. J. P. MAUNOIR, C. T. MAUNOIR, Professeurs F. MAYOR, C. G. PESCHIER, J. C. MORIN, J. P. DUPIN, F. OLIVET, Docteurs en Chirurgie.—Genève et Paris, 1824.

### NOTICE TO CORRESPONDENTS.

*Communications have been received from* Dr. Vandeburgh, Dr. Balfour, Mr. Hardy, Mr. Sanders, *&c. &c. which will appear in our ensuing Number.*

